# QTL Analysis and Candidate Gene Mapping for the Polyphenol Content in Cider Apple

**DOI:** 10.1371/journal.pone.0107103

**Published:** 2014-10-01

**Authors:** Cindy F. Verdu, Sylvain Guyot, Nicolas Childebrand, Muriel Bahut, Jean-Marc Celton, Sylvain Gaillard, Pauline Lasserre-Zuber, Michela Troggio, David Guilet, François Laurens

**Affiliations:** 1 INRA, UMR1345 Institut de Recherche en Horticulture et Semences, Beaucouzé, France; 2 Université d'Angers, UMR1345 Institut de Recherche en Horticulture et Semences, SFR 4207 QUASAV, PRES L'UNAM, Angers, France; 3 AgroCampus-Ouest, UMR1345 Institut de Recherche en Horticulture et Semences, Angers, France; 4 Université d'Angers, EA921 Laboratoire de Substances d'Origine Naturelle et Analogues Structuraux, SFR 4207 Quasav, PRES L'UNAM, Angers, France; 5 INRA, UR1268 Biopolymères, Interactions & Assemblages, Equipe « Polyphénols, Réactivité & Procédés », Le Rheu, France; 6 INRA-UBP, UMR1095 Genetics, Diversity and Ecophysiology of Cereals, Clermont-Ferrand, France; 7 Research and Innovation Centre, Fondazione Edmund Mach, S. Michele all'Adige, TN, Italy; Washington State University, United States of America

## Abstract

Polyphenols have favorable antioxidant potential on human health suggesting that their high content is responsible for the beneficial effects of apple consumption. They control the quality of ciders as they predominantly account for astringency, bitterness, color and aroma. In this study, we identified QTLs controlling phenolic compound concentrations and the average polymerization degree of flavanols in a cider apple progeny. Thirty-two compounds belonging to five groups of phenolic compounds were identified and quantified by reversed phase liquid chromatography on both fruit extract and juice, over three years. The average polymerization degree of flavanols was estimated in fruit by phloroglucinolysis coupled to HPLC. Parental maps were built using SSR and SNP markers and used for the QTL analysis. Sixty-nine and 72 QTLs were detected on 14 and 11 linkage groups of the female and male maps, respectively. A majority of the QTLs identified in this study are specific to this population, while others are consistent with previous studies. This study presents for the first time in apple, QTLs for the mean polymerization degree of procyanidins, for which the mechanisms involved remains unknown to this day. Identification of candidate genes underlying major QTLs was then performed *in silico* and permitted the identification of 18 enzymes of the polyphenol pathway and six transcription factors involved in the apple anthocyanin regulation. New markers were designed from sequences of the most interesting candidate genes in order to confirm their co-localization with underlying QTLs by genetic mapping. Finally, the potential use of these QTLs in breeding programs is discussed.

## Introduction

Apples can be separated in two main classes depending on their use: dessert and cider apples. The latter are generally bitter and astringent, more rustic, and many cider varieties are more resistant to the major pathogens of apple. Phenolic compounds are responsible for bitterness, astringency, color and may also partly contribute to aroma of cider. In relation to their tanning properties, procyanidins form aggregates with salivary proteins for astringency or receptors for bitterness, depending on their polymerization degree [1]. The color is linked to the enzymatic oxidation of phenolic compounds, mainly chlorogenic acid, procyanidins, (+)-catechin and phloridzin, by polyphenoloxydase [2]–[3]. Some hydroxycinnamic acids are also precursors of volatile phenols responsible for particular cider aroma that may be detrimental to the cider quality [4]. Apple consumption is inversely correlated with the development of diseases such as asthma, diabetes, cancer or cardiovascular diseases (for review see [5]–[6]). Their high phenolic content and antioxidant potential likely contribute to these protective effects. However, the mechanisms by which these compounds can exert this positive effect is still unclear (for review see [7]). In addition, interaction with other protective constituents such as dietary fibers plays a major role in the protection against these diseases [8]–[9].

Due to their importance in human health and their contribution to organoleptic properties, phenolic compounds have been characterized both qualitatively and quantitatively in whole fruit and various processed products like apple juice [10]–[14]. Globally, cider varieties are richer in total polyphenols than apple dessert varieties, with hydroxycinnamic acids, monomeric flavanols (*i.e.* catechins) and their oligomers and polymers (*i.e.* procyanidins and condensed tannins), dihydrochalcones and flavonols as the main phenolic groups.

**Figure 1:**
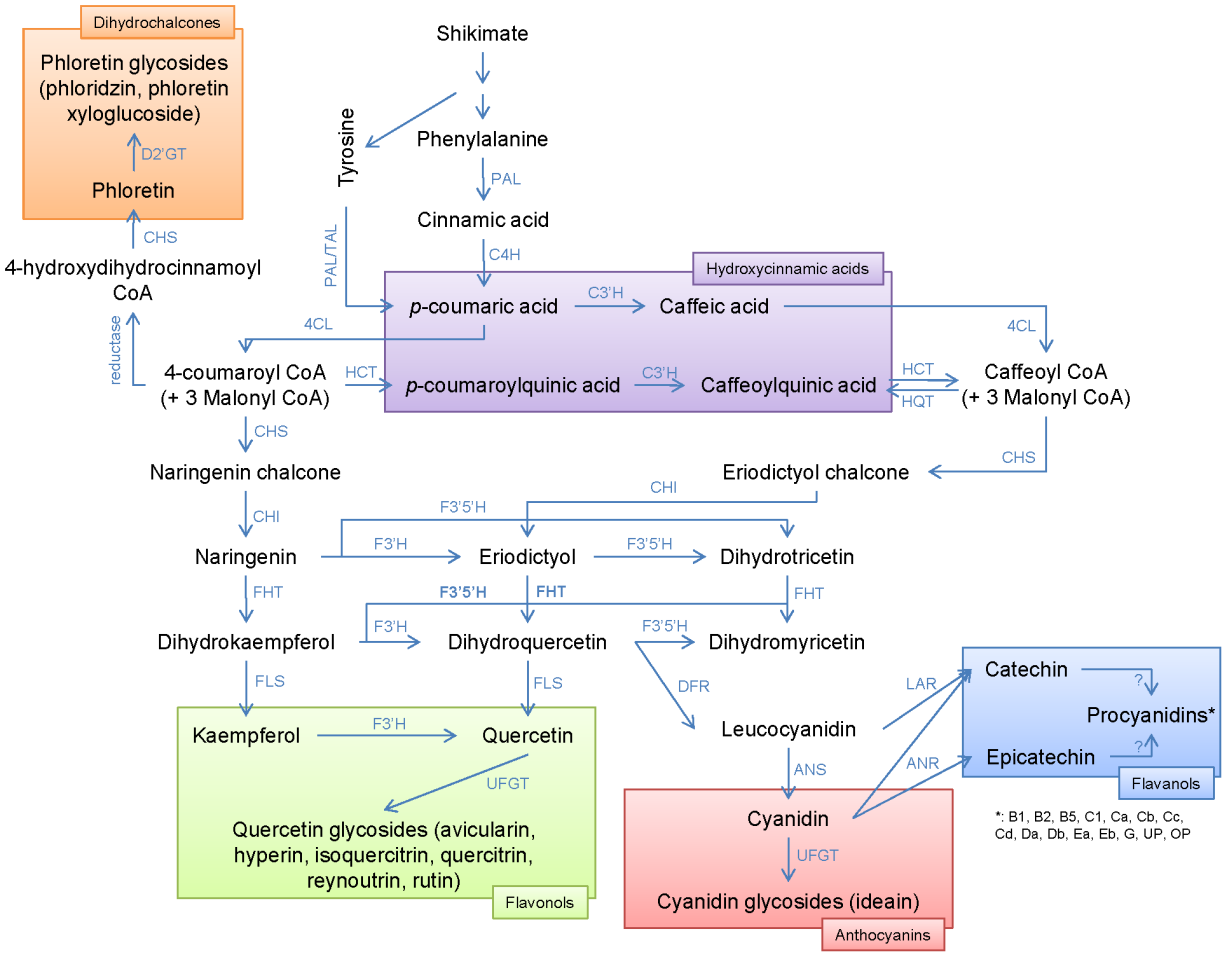
Phenolic compounds biosynthesis [15]–[20] (KEGG, 2012). In bold, enzymes identified in the support interval of QTLs. 4CL: 4-coumarate:CoA ligase; ANR: anthocyanidin reductase; ANS: anthocyanidin synthase; C3'H: *p*-coumarate 3′-hydroxylase; C4H: cinnamate 4-hydroxylase; CHI: chalcone isomerase; CHS: chalcone synthase; D2'GT: dihydrochalcone 2-*O*-glucosyltransferase; DFR: dihydroflavanol 4-reductase; F3'H: flavonoid 3′-hydroxylase; F3'5'H: flavonoid 3',5'-hydroxylase; FHT: flavanone 3-β-hydroxylase; FLS: flavonol synthase; HCT: shikimate *O*-hydroxycinnamoyl transferase; HQT: quinate *O*-hydroxycinnamoyl transferase; LAR: leucoanthocyanidin reductase; PAL: phenylalanine ammonia lyase; TAL: tyrosine ammonia lyase; UFGT: UDP-glucose 3-glucosyltransferase.

The polyphenol pathway is well known and several studies have highlighted the major enzymes involved, especially in *Arabidopsis* (Figure 1) [15]–[20]. However, mechanisms involved in the biosynthesis of procyanidins remain unknown [17]. Transcription factors of this pathway such as MYC [21]–[22], MYB [23]–[24], WD40-like protein [25], WRKY, MADS and TFIIIA-like protein (for review see [17]), activated by environmental stresses such as light [26], temperature [27] or wounding [23], have been identified in many species. So far in apple, three MYB (*MdMYB1* [28]/*MdMYBA* [29]/*MdMYB10* [30], *MYB110a* and *MYB110b* [31]), two bHLH (*MdbHLH3* [32] and *MdbHLH33* [30], [33]) and one WD40 (*MdTTG1*) [33] genes have been identified for their involvement in the anthocyanin pathway regulation.

QTL detection is a first step to detect genomic regions involved in phenotypic trait variation. Although widely investigated, phenolic compounds have been the subject of very few genetic studies. Only two studies have been recently published on the genetic basis of phenolic compound content of apple [34]–[35]. Both studies were performed on dessert apple progenies, separating skin from flesh, with two different approaches to quantify and map phenolic compounds. Chagné *et al*. reported the quantification of 16 and 23 phenolic compounds in two different harvest years using an ultra high performance liquid chromatography (UHPLC) coupled to a UV-PDA detector [34]. Seven clusters on linkage group (LG)1, LG14 and LG15 for *p*-coumaroylquinic acids, LG9 for anthocyanins, LG16 for flavanols, and LG17 for rutin and 5-caffeoylquinic acid were identified in this study. Khan *et al*. reported the quantification of 81 phenolic compounds belonging to the two groups of phenylpropanoids and polyphenols in the skin and the flesh, using a high performance liquid chromatography (HPLC) coupled to a mass spectrometer associated with MSClust software [35]. Five QTL hotspots on LG1 for quercetin and kaempferol glycosides, LG8 for quercitrin, LG13 for isorhamnetin glycosides, LG16 for kaempferol glycosides and flavanols, and LG17 for 5-caffeoylquinic acid were identified. In both studies, the top of LG16 was identified as the region controling the flavanol content variation. A candidate gene study revealed the presence of a *leucoanthocyanidin reductase* (*LAR*) gene, involved in flavanol biosynthesis, within the support interval of the LG16 QTLs detected both in flesh and skin [34]. None of these studies have included the mean polymerization degree (DPn) of procyanidins in their analyses.

For the first time in cider apples, this study aims to identify genomic regions controlling phenolic content by a QTL mapping approach. To achieve this goal, 32 phenolic compounds were quantified by liquid chromatography in fruit and juice prepared from a cider apple progeny, during three harvest years [36]. The DPn was also estimated by phloroglucinolyse reaction performed in fruit. For major QTL regions, candidate genes for phenolic content were identified *in silico* from the apple genome sequence, and their co-localizations were confirmed by genetic mapping.

## Material and Methods

### Plant material

No specific permission from the French regulatory authorities was required for this study. The location of this study is not protected in any way, and the study did not involve endangered or protected species.

Three hundred and eighty-five fruiting trees were analyzed in this study. They were derived from a cross between the two hybrids X5210 and X8402 produced at the Institut National de la Recherche Agronomique (INRA) cider apple breeding program (Figure S1). X5210 was derived from ‘Kermerrien’, a well-known French cider apple variety, while X8402 is a dessert apple hybrid derived from a cross between the varieties ‘Florina’ and ‘Prima’, selected for their resistance against apple pathogens. In the selection process, early selection tests for scab (*Venturia inaequalis*) and powdery mildew (*Podosphaera leucotricha*) resistance were performed in greenhouse and nursery respectively. Four hundred and sixty-two trees (29% of the population) remained after removing individuals susceptible to scab and powdery mildew and were planted on their own roots in 2003 in the orchard of the “Horticulture Experimental Unit” at INRA Angers-Nantes, France (47°28′39″N, 0°36′49″W).

Fruit extracts (flesh and skin together) were prepared from 92 hybrids harvested in 2008 and 137 harvested in 2009 (later denominated as F08 and F09, respectively). Apple juices (containing both flesh and skin) were prepared from 209 and 120 hybrids harvested in 2009 and 2010, respectively (later denominated as J09 and J10 respectively). All fruits were harvested from the tree at the mature stage corresponding to “50% of fallen fruits”, which is an easy visible metric employed in commercial cider orchards. This stage also has the advantage of providing comparable fruit maturity from one year to the next. The effect of light exposure was not recorded but it was minimized by sampling fruit (60 fruits per tree) in different parts of the tree (bottom/top; inside/outside) and by randomizing them before constituting each batch.

### Sample preparation and phenolic quantification

The quantification of phenolic compounds in fruit and juice was performed with a liquid chromatography system coupled with a UV-PDA detector and a mass spectrometer analyzer [36]. Compounds such as (+)-catechin, (−)-epicatechin, procyanidins B1 and B2, avicularin, hyperin, quercitrin, 5-caffeoylquinic acid, 4-*p*-coumaroylquinic acid and phloridzin were quantified each year and in both fruit and juice. Other compounds (procyanidins B5 and C1, 4-caffeoylquinic acid, isoquercitrin, reynoutrin, ideain, rutin and phloretin xyloglucoside) were only quantified in some experiments. Fourteen other compounds were quantified in J10: ten procyanidins, two unknown flavanols named by their molecular weight 245 and 518, the 5-*p*-coumaroylquinic acid and another phloretin xylohexoside (Table S1).

An acidolysis was performed on fruit extract to depolymerize procyanidins. This reaction allowed us to quantify all procyanidins present in the extract and estimate the mean polymerization degree of flavanols (DPnFlav, including monomers of flavanols) and procyanidins (DPnProc) [36]. The term “other procyanidins” (OP) includes all procyanidins of the extracts except procyanidins B1 and B2 quantified individually in fruit.

### DNA extraction and molecular marker genotyping

DNA extractions were performed from leaves using the CTAB extraction protocol as described by Tai and Tanksley [37] with some modifications: the leaves were ground in liquid nitrogen and 400 µl of the extraction buffer were added. The buffer was composed of 60 ml of the buffer A (0.35 M sorbitol, 0.1 M Tris-HCl, 5 mM EDTA), 25 ml of the buffer B (0.2 M Tris-HCl, 0.05 M EDTA, 2M NaCl, 2% CTAB), 10 ml of sarkosyl and 0.3 g of sodiumdisulfite. After the incubation at 65°C, 400 µl of chloroform were used instead of the potassium acetate. The incubation step on ice was removed.

One hundred and fifty three SSR markers available from HiDRAS project [38] and mapped by Silfverberg-Dilworth *et al*. [39] were amplified as described by Hibrand-Saint Oyant *et al*. [40] with some modifications: 0.22 µM of each primers and 2 ng of genomic DNA were used with the Qiagen multiplex PCR kit (Qiagen, Courtaboeuf, France). PCR amplifications were performed under the following conditions: initial denaturation at 94°C for 15 min followed by 34 cycles of 94°C for 30 sec, 55°C for 1 min 30 s, 72°C for 1 min. A final elongation step at 55°C for 15 min was included. PCR products were analyzed on a capillary sequencer 3730xl (Applied Biosystems, Saint Aubin, France) at the GENTYANE platform, INRA station of Clermont-Ferrand.

Three hundred and eighty four SNP markers identified in the ‘Golden Delicious’ genome sequence (Table S2) were genotyped using the Illumina Golden Gate assay at the GENTYANE platform according to the Illumina technology manufacturer's protocol.

### Genetic linkage map construction

Parental genetic linkage maps were built using JoinMap 4 software [41]. A logarithm of the odds (LOD) score threshold of five was used for grouping. Genetic distances between markers were calculated using Kosambi mapping function as described by Liebhard *et al*. [42].

### Statistical and QTL analysis

The broad sense heritability (h^2^) was calculated as follow: h^2^ = σ^2^_g_/(σ^2^_g_+σ^2^_r_) where σ^2^_g_ and σ^2^_r_ were the individual genetic and residual variances respectively.

QTL analysis was performed with the MapQTL 5 software on data obtained from the phenolic compound quantification each year on fruit and juice [43]. The DPn of flavanols and procyanidins estimated in fruit extract were also used in the analysis. Kruskal-Wallis (KW), Interval Mapping (IM) and multiple QTL mapping (MQM) functions were used to identify QTLs. Significance threshold was computed using a 1000 permutation tests, at the 95% genome-wide (GW) LOD thresholds. QTLs were described by their LOD score and their proportion of explained phenotypic variation (R^2^). The support intervals were defined as LOD-1 and LOD-2 around the maximum likelihood of QTL. The normality of residuals after QTL detection was calculated for each QTL [44] using a Shapiro-Francia normality test available in the ‘Nortest’ package of the R software version 3.0.3 [45]. MapChart software version 2.2 was used to represent confidence intervals of the mapped QTLs [46].

Epistatic effects were calculated for each trait for which more than one QTL was detected using a global model including all cofactors with the R software version 3.0.3 as described by Celton *et al*. [47].

### Candidate gene identification

All predicted gene sequences under the support interval of major QTLs were extracted from the apple genome sequence using tools based on the Bio++ libraries [48]. A blastx analysis was performed for each sequence on the non-redundant NCBI database with a minimum Blast Expected Value of 1.10^−3^ using the BLAST2GO software version 2.5.0 [49]. The KEGG pathway tool was used to select polyphenolic pathway enzymes [50]. The six transcription factors identified in apple for their anthocyanin pathway regulation (*MdMYB1*/*MdMYBA*/*MdMYB10*, *MYB110a*, *MYB110b*, *MdbHLH3*, *MdbHLH33*, *MdTTG1*) were also considered as putative candidate genes.

### Candidate gene mapping

PCR primers were designed based on contig sequence of the putative candidate genes. SSR were identified with the webstat software (http://wsmartins.net/webstat) and primers were designed with Primer3 software version 0.4.0. (http://frodo.wi.mit.edu/) using the same criteria as described by Chagné *et al*. [34] except for the final product size that ranged from 100 to 350 bp. Primer sequences are listed in Table 1. The M13 sequence was added at the 5′ position to analyze SSR products through a capillary sequencer as described by Hibrand-Saint Oyant *et al*. [40]. PCR products were analyzed on an ABI 3130xl genetic analyzer using the GeneScan 400HD ROX as size standard (Applied Biosystems, Saint Aubin, France). Electrophoregrams were analyzed with the GeneMapper 4.1 software and candidate genes were mapped to the parental maps using JoinMap 4 software.

**Table 1:** Properties of polymorphic SSR primers developed from ‘Golden Delicious’ genomic sequence for major candidate genes.

Gene^a^	LG	Contig	Expected size (bp)	Primer (5′-3′)	TM (°C)
F3’H*	1	MDC037600.14	155	F: AAGGGTAGGGCTAGAAGACACC	59
				R: ATAGATGTCGGCAACGTGAA	58
F3'5'H	1	MDC034541.5	159	F: TGGTCTTGGGTGCAAATCTG	61
				R: CCCTTCCCATTGATTCCTTC	60
FHT	1	MDC000829.287	169	F: GCGTGATTGGCTACGTGTAA	59
				R: TCGATTCGACTCTCGCACTA	59
MdTTG1	1	MDC001845.293	223	F: AATGAGACGAAATGTCCATCG	59
				R: CGGTGTTATCAGTTCACCAAAA	59
UFGT	1	MDC020818.61	249	F: CCAAAACCAAAGCATTCCAA	60
				R: GCATATTCGTGTTCTTGAAACC	58
DFR 1*	3	MDC012112.258	177	F: CTAGCCGAGTCAAACCAAGC	59
				R: ACTGCTGGTCCGAAAAGAAA	59
FHT/FLS*	5	MDC005520.264	225	F: CCTGAGTCTTGGGCACCTTA	59
				R: TTGGCACAAACGAGCAATAG	59
F3’H	5	MDC000463.206	183	F: CACATTGGTGGAGAATGGTG	59
				R: GCTTCCGTTCCAGCTAAGATT	59
CHI	12	MDC016452.88	224	F: CCACGGAGGAGTTTCTTGTC	59
				R: CATTCGGGTATCCTGCACTT	59
FLS*	12	MDC020724.428	218	F: GTTGGGCTGATGAAACTCGT	59
				R: AGTTGAATTTGGGCCTCCAT	60
F3'5'H	14	MDC011050.253	220	F: CACAGAGAATGGATGGGACA	59
				R: AACCACCTGCAATCAATCAC	58
CHS	15	MDC009830.388	292	F: TGGAGGACGAAGGAAATACG	59
				R: CAACGATGGCGTTCAAAAGT	60
MYB110a	17	MDC013323.290	240	F: CTCTCCCTCATCCCAGAACA	59
				R: TGCTGACTCCATTTCTTACTGC	59
MYB110b	17	MDC035405.20	212	F: CTTCGGGCTTATTTGGGTTT	60
				R: TTTGCCCCTTCAAAGATCAG	59
HCT/HQT 1	17	MDC015889.298	219	F: CTCCAAATGCAGATGAGGAA	58
				R: ATGGTGACTCCTACCGTCCA	60

*: enzymes which could not be mapped.

a : F3’H: flavonoid 3′-hydroxylase; F3'5'H: flavonoids 3',5'-hydroxylase; FHT: flavanone 3-β-hydroxylase; UFGT: UDP-glucose 3-glucosyltransferase; DFR: dihydroflavanol 4-reductase; FLS: flavonols synthase; CHI: chalcone isomerase; CHS: chalcone synthase; HCT/HQT: shikimate/quinate hydroxycinnamoyltransferase.

## Results

### Construction of genetic maps

Of the 153 SSR markers tested, 80 (52.3%) and 64 (41.8%) were used, for the female and male linkage map construction, respectively. Of the 384 SNP markers, 170 (44.3%) were monomorphic, 30 (7.8%) did not amplify or were unreadable and 184 (47.9%) were polymorphic (98 abxaa, 57 aaxab, 29 abxab). Among the 184 polymorphic SNP markers, only 54 and 45 were kept for the female and male map construction, respectively. The female map covered 1191.6 cM over 16 LG and the male map covered 1005.3 cM over 17 LG (Figure S2). Only one SNP marker was polymorphic for the female LG7. Distortions were observed on the LG1, LG11 and LG17 of both parental maps.

### QTL detection

The individual broad sense heritability was higher for compounds quantified in fruit extract than for those quantified in apple juice (Table 2). Broad sense heritability values ranged from 0.13 to 0.98. Only heritabilities of avicularin and hyperin were less than 0.5 (h^2^ = 0.13 for avicularin in J09 and h^2^ = 0.39 for hyperin in J10).

**Table 2:** Broad sense genetic heritability of mean polymerization degree and phenolic compounds quantified in fruits harvested in 2008 (F08) and 2009 (F09) and in juices prepared in 2009 (J09) and 2010 (J10).

Heritability	F08^b^	F09^b^	J09^b^	J10^b^
(−)-epicatechin	0.96	0.94	0.80	0.61
(+)-catechin	0.97	0.77	0.79	0.79
procyanidin B1	0.98	0.96	0.78	0.79
procyanidin B2	0.95	0.89	0.84	0.93
other procyanidins	0.96	0.92	na	na
DPn flavanols^a^	0.89	0.96	na	na
5-caffeoylquinic acid	0.92	0.95	0.87	0.77
4-caffeoylquinic acid	na	na	0.80	0.87
4-p-coumaroylquinic acid	0.96	0.93	0.88	0.80
avicularin	0.58	0.57	0.13	0.59
hyperin	0.60	0.62	0.57	0.39
isoquercitrin	0.80	0.65	na	na
quercitrin	0.88	0.74	0.70	0.68
reynoutrin	0.71	0.68	na	na
rutin	0.72	na	0.52	0.56
phloridzin	0.90	0.89	0.83	0.79
phloretin xyloglucoside	0.88	0.92	na	0.75
ideain	na	0.77	na	na

a : DPn: mean polymerization degree.

b : na: not available.

Sixty nine and 72 QTLs were detected at GW threshold on 14 and 11 LGs of the female and male maps, respectively, for the DPn and all phenolic compounds, except phloretin xylohexoside. Table 3 shows results for both KW and MQM (LOD and R^2^) analyses, since MQM detection is sufficiently robust with non-normally distributed traits [51]. Results are similar with both parametric and non-parametric tests.

**Table 3:** Quantitative trait loci (QTL) detected in the apple X5210 and X8402 parental genetic maps by multiple QTL mapping (MQM) analysis and Kruskal-Wallis (KW) test for phenolic compounds and the mean polymerization degree of flavanols estimated in fruits harvested in 2008 and 2009 and in juices prepared in 2009 and 2010.

Compound	Material	Year	Parent	Linkage group	LOD score^a^	% variation^b^	Marker with highest LOD	Residuals normality^c^	Epistatic effects^d^	KW	Marker with highest KW
**FLAVANOL MONOMERS**											
Catechin	Juice	2009	X5210	12	5.27	10.8	GD_SNP02857	<0.01		7	
	Juice	2009	X8402	1	2.83	6.3	GD_SNP00087	<0.01	-	4	
	Juice	2009	X8402	15	3	7.4	Hi02d02			7	
	Juice	2010	X5210	3	2.66	8.6	GD_SNP01990	<0.01	-	2	
	Juice	2010	X5210	10	3.01	10	Ch02a08			4	
	Juice	2010	X5210	17	3.22	11.8	Hi02f12			2	
	Juice	2010	X8402	4	1.92	6.4	NZ05g08	<0.01	-	4	
	Juice	2010	X8402	15	6.12	20.3	Ch02d11			7	
Epicatechin	Fruit	2009	X5210	12	3.72	9.5	GD_SNP02857	<0.01		6	
	Juice	2009	X5210	6	4.19	8.9	Ch03d07	<0.01		6	
	Juice	2009	X8402	1	4.39	8.6	GD_SNP01678	<0.01	-	6	
	Juice	2009	X8402	12	5.01	10.8	Ch01g12			7	
	Juice	2009	X8402	14	3.47	7	Ch01g05			4	
	Juice	2010	X5210	15	2.9	9.8	Hi03g06	0.08	-	4	
	Juice	2010	X5210	16	2.86	10.5	Ch05c06			5	GD_SNP00923
	Juice	2010	X8402	1	4.28	19.4	GD_SNP00087	0.1		6	
**PROCYANIDINS**											
Procyanidin B1	Fruit	2008	X8402	1	3.24	15.3	GD_SNP01678	0.04		6	
	Fruit	2009	X5210	12	5.6	16.5	GD_SNP02857	0.07	-	7	Ch05d11
	Fruit	2009	X5210	16	2.79	8.9	Ch05c06			3	
	Fruit	2009	X8402	17	4.08	12	Hi02f12	<0.01		4	AT000174
	Juice	2009	X5210	3	3.34	5.8	MS14h03	<0.01	* MS14h03:SNP02857 *** MS14h03:Ch02a08	5	
	Juice	2009	X5210	6	3.45	5.8	Ch03d07			2	
	Juice	2009	X5210	10	2.5	4.3	Ch02a08			4	
	Juice	2009	X5210	12	6.88	12.2	GD_SNP02857			7	
	Juice	2009	X8402	1	3.42	7.2	GD_SNP00087	<0.01	-	6	
	Juice	2009	X8402	15	2.84	7.5	Hi02d02			7	
	Juice	2010	X5210	16	3.17	10.2	GD_SNP00626	<0.01		2	
	Juice	2010	X8402	1	4.09	12.6	GD_SNP01678	<0.01	-	7	
	Juice	2010	X8402	4	3.11	8.8	NZ05g08			4	
	Juice	2010	X8402	15	3.27	9.1	Ch03b10			7	
Procyanidin B2	Fruit	2008	X5210	1	3.53	19.1	HB11AG	0.76		4	
	Fruit	2009	X5210	1	4.42	16.7	GD_SNP01772	<0.01		7	HB11AG
	Fruit	2009	X8402	1	3.07	11.1	GD_SNP01678	<0.01		6	Ch05g08
	Juice	2009	X5210	3	5.4	10.4	Hi03d06	<0.01		7	
	Juice	2009	X8402	1	7.32	15.7	GD_SNP01678	<0.01	-	7	
	Juice	2009	X8402	15	2.93	6.1	Ch02d11			2	
	Juice	2010	X8402	1	5.3	19.4	GD_SNP00252	<0.01		7	
Procyanidin B5	Juice	2009	X5210	3	7.66	15.3	Hi03d06	<0.01		7	
	Juice	2009	X8402	1	7.77	15.5	GD_SNP01678	<0.01	-	7	
	Juice	2009	X8402	7	3.15	7.7	Hi05b09			3	
	Juice	2010	X5210	8	2.93	10.5	Hi04b12	<0.01		5	
	Juice	2010	X8402	1	5.69	20.4	GD_SNP00087	<0.01		7	
Procyanidin C1	Juice	2009	X5210	3	3.46	6	Hi03d06	<0.01	-	7	
	Juice	2009	X5210	12	3.98	7.7	Ch01g12			7	
	Juice	2009	X8402	1	6.61	13.8	GD_SNP01678	<0.01		7	
	Juice	2010	X5210	15	2.95	10.3	Ch05a02y	<0.01		4	
	Juice	2010	X8402	1	5.22	23	GD_SNP00087	0.01		7	
Procyanidin Ca	Juice	2010	X5210	3	2.68	7.5	Hi03d06	<0.01	-	2	
	Juice	2010	X5210	12	2.97	8.3	Ch01g12			4	
	Juice	2010	X8402	1	2.52	8.1	GD_SNP01678	<0.01	* Ch02d11:NZ05g08	7	
	Juice	2010	X8402	4	3.3	10.2	NZ05g08			5	
	Juice	2010	X8402	15	4.35	12.2	Ch02d11			7	
Procyanidin Cb	Juice	2010	X8402	1	4.11	13.3	GD_SNP01678	<0.01	-	7	
	Juice	2010	X8402	15	2.68	9	Ch02d11			7	Ch03b10
Procyanidin Cc	Juice	2010	X5210	11	2.74	8.3	GD_SNP00662	<0.01		4	
	Juice	2010	X8402	4	3.06	9.6	NZ05g08	0.015	-	6	
	Juice	2010	X8402	15	5.59	18	Ch02d11			7	
Procyanidin Cd	Juice	2010	X5210	6	3.89	10.6	Ch03d07	0.56	-	4	
	Juice	2010	X5210	12	3.43	9.2	Ch01g12			5	
	Juice	2010	X5210	15	3.04	8.1	Hi03g06			4	
	Juice	2010	X8402	15	3.5	13.1	Ch03b10	0.8		7	
Procyanidin Da	Juice	2010	X8402	4	2.98	9.7	NZ05g08	0.012	-	4	
	Juice	2010	X8402	15	6.37	17.6	Ch02d11			7	
Procyanidin Db	Juice	2010	X8402	1	3.62	15.4	GD_SNP00087	0.07		6	
Procyanidin Ea	Juice	2010	X8402	1	4.37	11.2	GD_SNP01678	0.16	-	7	
	Juice	2010	X8402	15	6.71	18.9	Ch02d11			7	
Procyanidin Eb	Juice	2010	X8402	1	5.12	21.2	GD_SNP01678	0.1		6	GD_SNP00252
Procyanidin G	Juice	2010	X5210	9	2.86	7.3	Ch01f03b	0.02	* Ch01f03b:Hi03g06	2	
	Juice	2010	X5210	12	3.24	8.4	Ch01g12			4	
	Juice	2010	X5210	15	3.07	8	Hi03g06			4	
Other procyanidins	Fruit	2008	X5210	5	5.09	22.5	Ch03a04	0.15	-	7	
	Fruit	2008	X5210	15	3.2	12.9	Ch05a02y			3	
	Fruit	2008	X8402	1	4.32	18.8	GD_SNP01678	0.014	-	7	
	Fruit	2008	X8402	13	4.64	30	NH009b			5	
	Fruit	2009	X5210	1	3.64	12.2	Ch05g08	<0.01		6	
	Fruit	2009	X8402	1	2.89	9	Ch05g08	<0.01		6	
Unknown Procyanidin	Juice	2010	X8402	1	6.68	22.7	GD_SNP00087	<0.01		7	
**MEAN POLYMERIZATION DEGREE**											
DPn Flavanols	Fruit	2008	X5210	5	3.6	13.8	Ch03a04	0.81	-	3	
	Fruit	2008	X5210	16	5.51	22.7	Ch05c06			7	
	Fruit	2008	X8402	15	3.76	16.2	Ch02d11	0.01		6	
DPn Procyanidins	Fruit	2008	X5210	12	4.6	20.1	Hi07f01	0.86	-	2	
	Fruit	2008	X5210	14	2.95	12.7	U78948-SSR			2	
	Fruit	2008	X5210	16	5.95	23.6	Ch05c06			6	
	Fruit	2008	X8402	1	3.12	16	HB11AG	0.04		6	
**UNKNOWN FLAVANOLS**											
Unknown flavanol 245	Juice	2010	X5210	15	3.22	10.4	Ch02d11	<0.01		3	
	Juice	2010	X8402	1	4.72	13.4	GD_SNP00087	<0.01	-	6	
	Juice	2010	X8402	7	3.33	10.6	GD_SNP01040			3	
Unknown flavanol 518	Juice	2010	X5210	15	3.12	9.5	Hi03g06	0.37		4	
**HYDROXYCINNAMIC ACIDS**											
4-caffeoylquinic acid	Juice	2009	X5210	14	1.82	4	GD_SNP00207	<0.01		7	
	Juice	2010	X5210	14	9.27	34.7	Ch05g07	<0.01		7	
	Juice	2010	X8402	1	3.12	12.4	Ch05g08	<0.01		6	
4-*p*-coumaroylquinic acid	Fruit	2008	X5210	14	7.97	31.5	GD_SNP00207	0.05		7	
	Fruit	2009	X5210	14	8.71	28.5	GD_SNP00207	<0.01		7	
	Juice	2009	X5210	12	3.12	6.1	GD_SNP02857	<0.01	-	2	
	Juice	2009	X5210	14	9.07	15.9	GD_SNP00207			7	
	Juice	2009	X8402	15	1.58	4.1	GD_SNP00273	<0.01		4	
	Juice	2010	X5210	14	14.27	46.5	GD_SNP00207	<0.01		7	
	Juice	2010	X8402	1	4.22	13.6	Ch05g08	<0.01		7	
5-caffeoylquinic acid	Fruit	2008	X5210	17	5.26	23.2	Hi02f12	0.14		7	
	Fruit	2008	X8402	17	5.63	23.3	Hi02f12	0.3		7	
	Fruit	2009	X5210	17	5.61	20.3	Hi02f12	<0.01		7	
	Fruit	2009	X8402	17	6.15	25.4	Hi02f12	0.2		7	
	Juice	2009	X5210	3	3.05	7.1	GD_SNP01969	0.03	-	6	
	Juice	2009	X5210	17	7.62	16.4	Hi02f12			7	
	Juice	2009	X8402	17	11.64	26.8	Hi02f12	0.4		7	
	Juice	2010	X5210	17	6.84	25.7	Hi02f12	0.02		7	
	Juice	2010	X8402	17	6.07	19	Hi02f12	<0.01		7	
5-*p*-coumaroylquinic acid	Juice	2010	X5210	17	3.02	12.3	Hi02f12	<0.01		6	
	Juice	2010	X8402	1	3.72	11.9	Ch05g08	<0.01		7	
**FLAVONOLS**											
Avicularin	Fruit	2008	X5210	15	3.8	19.1	Ch02d11	0.38		6	
	Juice	2009	X5210	5	3.12	6.4	Ch05gf06	<0.01		4	
Hyperin	Fruit	2008	X5210	15	4.32	20	Ch02d11	0.02		4	
	Juice	2009	X5210	15	3.19	6.7	Ch03b10	<0.01		4	
	Juice	2009	X8402	16	3.26	8.5	Ch02d10a	<0.01		5	
	Juice	2010	X5210	15	3.17	14	Ch02d11	<0.01		4	
Isoquercitrin	Fruit	2008	X5210	15	2.76	17.8	Ch02d11	<0.01		3	
	Fruit	2009	X8402	5	2.6	11.8	Ch05f06	<0.01	* (GD_SNP00256:Hi02f12)	2	
	Fruit	2009	X8402	7	3.36	15.3	GD_SNP00256			5	
	Fruit	2009	X8402	17	3.22	11.8	Hi02f12			4	
Quercitrin	Fruit	2009	X5210	13	3.35	12.1	Hi04f09	<0.01		7	
	Fruit	2009	X8402	17	3.13	12	Hi03c05	0.02		6	
	Juice	2009	X8402	10	3.12	6.3	Ch02c11	<0.01		2	
	Juice	2010	X5210	13	4.03	15.1	Hi04f09	<0.01		6	
	Juice	2010	X8402	1	5.1	19.2	GD_SNP00087	<0.01		7	
Reynoutrin	Fruit	2008	X5210	15	3.6	17.3	Ch02d11	0.08		4	
	Fruit	2008	X8402	14	3.55	26	Ch01g05	0.07	**	2	
	Fruit	2008	X8402	16	2.93	11.9	Ch05a04			3	
	Fruit	2009	X5210	15	4.1	15.6	Ch02d11	<0.01		7	
	Fruit	2009	X8402	1	3.14	13.4	Hi02c07	<0.01		5	
Rutin	Fruit	2008	X8402	17	2.67	15	Ch04f08	<0.01		5	
	Juice	2009	X5210	8	2.75	5.7	Ch01c06	<0.01		2	Ch01f09
**DIHYDROCHALCONES**											
Phloretin xyloglucoside	Fruit	2008	X8402	5	2.7	19.5	GD_SNP00231	0.16	-	3	
	Fruit	2008	X8402	12	3.73	21.2	GD_SNP00762			6	
	Fruit	2009	X8402	5	5.04	17.3	GD_SNP00632	<0.01		7	
	Juice	2010	X5210	3	5.14	17.4	GD_SNP01969	0.04	-	4	AU223657
	Juice	2010	X5210	15	6.87	23.6	GD_SNP01146			6	Ch05a02y
Phloridzin	Fruit	2008	X8402	1	3.07	11.6	Ch05g08	<0.01		7	
	Juice	2009	X8402	5	2.67	6.4	GD_SNP00632	<0.01		4	
	Juice	2010	X8402	1	3.49	11.3	GD_SNP01678	<0.01		5	Ch05g08
**ANTHOCYANINS**											
Ideain	Fruit	2009	X5210	1	3.36	19.2	HB11AG	<0.01		4	
	Fruit	2009	X8402	5	2.36	12.4	GD_SNP00189	<0.01		4	Ch05e06

a : Maximum logarithm of the odds (LOD) scores value of the QTL.

b : Proportion of the phenotypic variation explained by the QTL.

c : results of the Shapiro-Francia normality test calculated with the residuals obtained after QTL detection.

d : *, P = 0.1; **, P = 0.05; ***, P = 0.01.

#### Flavanols and their mean polymerization degree (DPn)

Six clusters of QTL were detected on LG3, LG6, LG10, LG12, LG15 and LG16 on the female map (X5210) and four clusters on LG1, LG4, LG7 and LG15 on the male map (X8402) with a proportion of explained phenotypic variation comprised between 6 to 30% (Table 3). Clusters on LG1, LG12 and LG16 were identified for many flavanols detected in both fruit and juice independent of the harvest year. QTLs on LG3, LG4, LG6, LG7, LG10 and LG15 were specifically detected for compounds quantified in juice (Table 3). Epistatic effects were significant between QTLs on LG3 and LG10 and between LG3 and LG12 for the procyanidin B1 quantified in J09. Epistatic effects were also significant between QTLs on LG1, LG4 and LG15 of the male map of the procyanidin Ca and between QTLs on LG9, LG12 and LG15 of the female map of the procyanidin G (Table 3).

Five QTLs were detected for the DPnFlav and DPnProc estimated in F08 on LG5, LG12, LG14 and LG16 of the female map, explaining between 13 and 24% of the phenotypic variation. Two other QTLs were detected on the male map in F08 on LG1 and LG15, explaining 16% of the phenotypic variation (Table 3).

#### Hydroxycinnamic acid

A QTL was detected for hydroxycinnamic acids esterified in fourth position (4-caffeoylquinic and 4-*p*-coumaroylquinic acids) at the top of LG14 on the female map (X5210), explaining between 4 and 46% of the phenotypic variation (Table 3). We identified a QTL for 5-caffeoylquinic acid content for fruit and juice on the LG17 of both parental maps. The proportion of phenotypic variation explained by this QTL varied from 16 to 26% (Table 3). A cluster of QTLs was detected for hydroxycinnamic acids quantified in J10 at the bottom of the LG1 of the male map grouping QTLs for the 4-caffeoylquinic acid, the 4-*p*-coumaroylquinic acid and the 5-*p*-coumaroylquinic acid, explaining between 12 and 14% of the phenotypic variation (Table 3).

#### Flavonols

A major region controlling flavonols concentration was localized at the bottom of the female (X5210) LG15 at the same position as the flavanol cluster detected on the male map (X8402). Seven QTLs were detected for avicularin, hyperin, isoquercitrin and reynoutrin quantified each year in both fruit and juice, with a proportion of explained phenotypic variation comprised between 3 and 20% (Table 3). Another region was detected for quercitrin quantified in F09 and J10 on the LG13 of the female map, with two QTLs explaining 12 and 15% of the phenotypic variation. Significant epistatic effects were found for isoquercitrin quantified in F09 between QTLs detected on LG7 and LG17 and for reynoutrin quantified in F08 between QTLs detected on LG14 and LG16 (Table 3).

#### Dihydrochalcones

Two clusters of QTLs were detected on the LG1 for phloridzin and on the LG5 for phloridzin and phloretin xyloglucoside on the male map (X8402), and explained between 6 and 19% of the phenotypic variation (Table 3). Single QTLs for phloretin xyloglucoside quantified in F08 and J10 were detected on LG12 of the male map and on LG3 and LG15 of the female map, explaining 21, 17 and 24% of the phenotypic variation respectively.

#### Anthocyanins

Two QTLs were detected for ideain concentration on the LG1 female map (X5210) and on the LG5 male map (X8402), explaining 19 and 12% of the phenotypic variation, respectively (Table 3).

### Putative candidate genes identification

Genomic regions located on LG1, LG3, LG5, LG12, LG14, LG15 and LG17 underlying QTLs for flavanols, dihydrochalcones, flavonols and hydroxycinnamic acid were selected for candidate gene identification. Concerning the hydroxycinnamic acids pathway, three genes homologous to *flavonoid 3′-hydroxylase* (*F3'H*) and two homologous to *flavonoids 3',5'-hydroxylase* (*F3'5'H*) were identified on LG14. Four genes annotated as *shikimate/quinate hydroxycinnamoyltransferase* (*HCT/HQT*) and as *dihydroflavanol 4-reductase* (*DFR*) were identified on LG17 (Table 4). The *MYB110a* and *MYB110b* genes were also identified within the same interval on LG17. For flavanols, one gene with sequence homology to *UDP-glucose 3-glucosyltransferase* (*UFGT*), three genes homologous to *flavonoid 3′-hydroxylase* (*F3'H*) and one homologous to *flavonols synthase* (*FLS*) were identified on LG1. The *MdTTG1* transcription factor was also identified within this interval. Four genes annotated as *DFR*, two *F3'5'H* and one *FLS* were found on LG3. On LG12, two *FLS* were also identified. The *bHLH33* transcription factor was localized under the cluster of flavonols on the LG15. One *F3'H* and one *F3'5'H* were identified for flavonols on LG1. For dihydrochalcones, six genes annotated as *UFGT*, *F3'H*, *FHT*, *DFR* and *FLS* were identified on LG5, one *chalcone isomerase* (*CHI*) was identified on LG12 and one *chalcone synthase* (*CHS*) and one *FLS* were identified on LG15.

**Table 4:** Selected candidate genes identified in the interval of 12 clusters of quantitative trait loci (QTL) using the BLAST2GO software.

Phenolic group	LG	Top marker	Down marker	Number of sequences	Putative gene functions^a^	MDP^b^	Length (bp)^c^	# hits^d^	Min e.Value^e^	sim. Mean (%)^f^
Hydroxycinnamic acid	14	GD_SNP00207	Ch05g07	1066	F3'H	MDP0000261732	714	20	4.10e^−73^	82.85
						MDP0000443803	177	20	7.10e^−14^	86.25
						MDP0000848416	381	20	2.40e^−43^	89.55
					F3'5'H	MDP0000285273	1590	20	0	77.5
						MDP0000308262	1770	20	0	77.05
	17	Ch04f08	Hi07h02	1438	HCT/HQT	MDP0000153269	197	20	1.5e^−12^	82.25
						MDP0000261618	348	20	6.5e^−26^	72.5
						MDP0000307780	1538	20	0	89.35
						MDP0000371737	1326	20	0	83.2
					DFR	MDP0000648997	1515	20	4.80e^−127^	78.9
					MYB110a	MDP0000295218	GenBank accession number EB710109			
					MYB110b	MDP0000317257	GenBank accession number CN993940			
Flavanols	1	GD_SNP00183	Ch05g08	2501	UFGT	MDP0000478252	1452	20	0	79.4
					MdTTG1		GenBank accession number GU173813			
					F3'H	MDP0000214162	999	20	1.5e^−178^	81.15
						MDP0000416305	1026	20	0	84.75
						MDP0000657536	453	20	3.7e^−84^	87.35
					FLS	MDP0000155229	990	20	1.5e^−142^	64.9
	3	Hi03d06	GD_SNP01969	2000	DFR	MDP0000204525	1572	20	0	92.15
						MDP0000265073	1626	20	0	92.3
						MDP0000268045	1020	20	0	92
						MDP0000729984	978	20	0	92.2
					F3'5'H	MDP0000630030	1383	20	0	78.05
						MDP0000780878	408	20	3.4e^−24^	80.15
					FLS	MDP0000703138	270	20	2.7e^−25^	78.4
	12	Ch05d11	Ch01g12	963	FLS	MDP0000390769	270	20	2.7e^−25^	78.4
						MDP0000041421	1116	20	0	76.65
	15	Ch02d11	GD_SNP02455	1021	MdbHLH33		GenBank accession number DQ266451			
Dihydrochalcones	5	Ch04g09x	GD_SNP00231	1296	UFGT	MDP0000708060	369	20	1.20e^−63^	83.85
					F3'H	MDP0000692178	1866	20	0	70.55
					FHT	MDP0000515855	1089	20	0	65.1
					DFR	MDP0000414002	921	20	4.40e^−148^	77.15
					FLS	MDP0000222546	1500	20	1.50e^−29^	77.25
						MDP0000515855	1089	20	0	65.1
	12	Ch03c02	Hi07f01	1132	CHI	MDP0000252589	732	20	1.30e^−93^	88.5
	15	Hi03g06	Hi02d02		CHS	MDP0000287919	1167	20	0	83.7
					FLS	MDP0000159118	258	20	1.90e^−15^	81.1
Flavonols	1	GD_SNP01470	GD_SNP00183	658	F3'H	MDP0000140803	1611	20	0	80.35
					F3'5'H	MDP0000675937	2556	20	0	86.85
	15	Ch02d11	GD_SNP02455	1021	MdbHLH33		GenBank accession number DQ266451			

a : CHI: chalcone isomerase; CHS: chalcone synthase; DFR: dihydroflavanol 4-reductase; F3'H: flavonoid 3′-hydroxylase; F3'5'H: flavonoids 3',5'-hydroxylase; FLS: flavonols synthase; HCT/HQT: shikimate/quinate hydroxycinnamoyl transferase; UFGT: UDP-glucose 3-glucosyltransferase.

b : contig containing the gene on the apple genome browser.

c : longest hits which aligned with the sequence.

d : numbered of alignment achieved.

e : estimator of the quality of the alignment.

f : average proportion of sequence similarity.

**Figure 2:**
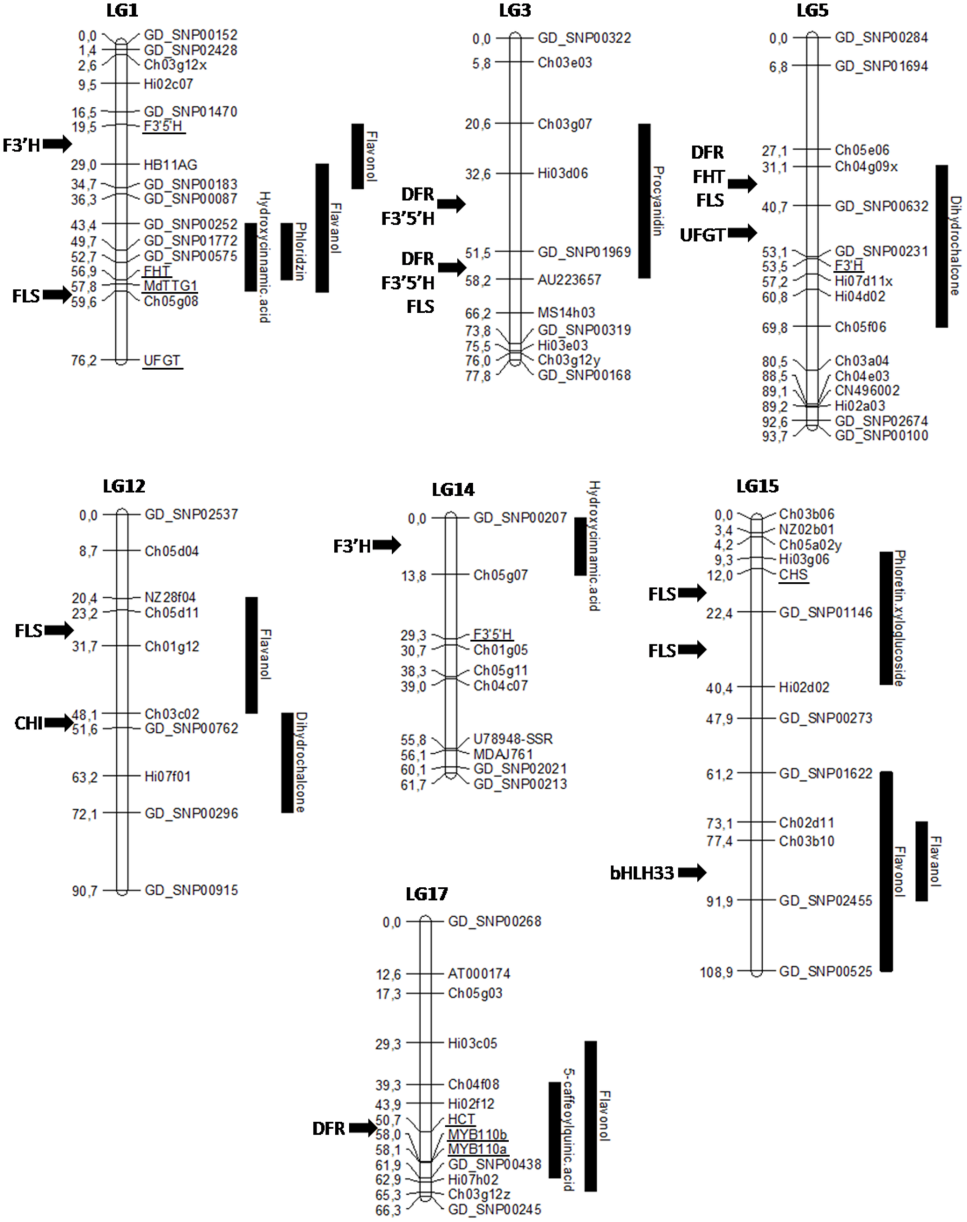
Main interesting clusters of quantitative trait loci (QTL) for phenolic compounds in fruit and juice. Main QTL clusters obtained are represented with black bars on the right of the corresponding linkage groups (LG). Putative candidate genes identified *in silico* and their relative position on the map are specified on the left of the LG. Genetically mapped candidate genes are indicated on the right of the LG and underlined.

Fifteen pairs of primers were designed to map the candidate genes (Table 1). Eleven amplified and were polymorphic in the X5210×X8402 progeny. Of these, eight were mapped at the predicted position. The genes homologous to *F3'5'H*, *FHT* and *MdTTG1* genes were mapped on LG1, the *F3'H* apple homologue on LG5, the *F3'5'H* apple gene homologue on LG14, the gene homologous to *CHS* on LG15 and the *HCT/HQT* apple homologues, *MYB110a* and *MYB110b* genes on LG17 (Figure 2). Of the three others, one failed to map (*FLS*), while two mapped outside of the *in silico* expected regions (*UFGT* expected between GD_SNP00252 and Ch05g08, and *CHI* expected between GD_SNP00762 and Hi07f01).

## Discussion

This study was performed with a breeding population designed for the selection of cider apple varieties. The cross of a cider with a dessert apple hybrid has maximized the diversity and could explain the higher number of QTLs detected compared with previous studies. The grandparent ‘Kermerrien’ was selected for its cider quality and the dessert ancestors of the progeny were selected for their resistance to scab and/or powdery mildew and carry some resistance genes e.g. *Vf* on the LG1 [52] and *Pl2* on the LG11 [53], respectively. These genes were transmitted to the progeny and the early selection made for the resistance to both pathogens could explain distortions observed on linkage maps. For the QTL detection, biases caused by distortions were overcome by performing permutation tests to determine a LOD threshold for each linkage group. Moreover, the selection for scab and powdery mildew resistance did not affect the QTL detection of phenolic compounds except in strongly distorted regions (on LG1, LG11 and LG17). Therefore, it is possible that the pre-selection of seedlings may have reduced the number of QTLs detected in the analysis.

### Marker polymorphism and genetic map construction

The proportion of SSR markers polymorphic for X5210 and X8402 (52.3 and 41.8%, respectively) was comparable with previous study [54]. The proportion between monomorphic, not amplified or unreadable, and polymorphic SNP markers was similar to that of Micheletti *et al*. with a transferability rate of 40.9% between different apple cultivars [55]. Among the 184 polymorphic SNP markers, 48% were kept for the parental map construction, with sizes comparable to reference maps [39], [42], [54].

### New QTL identified and validation from previous studies

Our study reports for the first time on new genetic regions controlling the mean polymerization degree (DPn) of procyanidins in apple. QTLs were detected on the LG12, LG14 and LG16 on the female map and on the LG1 and LG15 on the male map (Table 3). Only those on LG15 and LG16 co-localized with QTLs for the flavanol content, supporting an independent regulation for the flavanol concentration and the flavanol polymerization in apple. The polymerization degree of flavanols is particularly interesting for apple since it modifies organoleptic properties of procyanidins: low DPn (<5) affect the bitterness of apple, while high DPn (6<DPn<10) affect the astringency [1]. Moreover, molecular and genetic mechanisms involved in the synthesis of procyanidins remain unknown [17]. This study is therefore a first step opening new perspectives to understand mechanisms implied in the polymerization of flavanols.

Moreover, this study has identified many QTLs for flavanols and dihydrochalcones not yet detected in previous studies in dessert apple. Nine main regions were detected for the flavanol content on LG3, LG6, LG10, LG12, LG15 and LG16 of the female map and on LG1, LG4, LG7 and LG15 of the male map. In Chagné *et al*. [34] and Khan *et al*. [35] studies, the most important QTLs for the flavanol content was detected at the top of the LG16 (above the cluster detected on the female map), where a gene homologous to *leucoanthocyanidin reductase* (*LAR*) was identified in the support interval. This region was not detected in our study. We suppose that the parents of our population were homozygous for this QTL, which may explain the fact that we did not identify it during the QTL analysis. Since no marker distortion was observed at the top of this LG, we can affirm that the important selection performed on the population is not responsible for the absence of this QTL. For dihydrochalcones, several QTLs were detected in the X5210×X8402 progeny on LG1, LG3, LG5, LG12 and LG15. In previous works, only two QTLs were identified by Khan *et al*. for phloridzin quantified in skin (on LG16) and flesh (on LG15) [35].

The comparison with previous works published by Chagné *et al*. [34] and Khan *et al*. [35] has also shown two stable and conserved regions across studies for the 4-*p*-coumaroylquinic and 4-caffeoylquinic acids on LG14 and the 5-caffeoylquinic acid on LG17. For the QTLs on LG17, the proportion of explained phenotypic variation was particularly high in all three surveys (19% in Khan *et al*. study, from 10 to 46% in Chagné *et al*. study, and from 16 and 27% in our study).

To recap, this study has identified 23 new regions for the DPn, flavanols, flavonols, dihydrochalcones and anthocyanins content and confirmed five regions, three for hydroxycinnamic acids on LG1, LG14 and LG17, one for dihydrochalcones on LG15, and one for flavonols on LG17.

### Comparison between fruit and juice

QTL detection performed in fruit and juice yielded similar result for all classes of phenolic compounds except for flavonols: there are more QTL detected in fruit than in juice. Previous studies have shown that the phenolic content of cider is greatly dependent on the environmental conditions in which apple trees grow [56]–[57] and the conditions of fruit storage and pressing [36], [58]. In this study, the lowest extractability observed for flavonols [36], mainly explain the higher number of QTLs detected in fruit than in juice.

For flavanols, the higher number of regions detected in juice is due to the higher number of procyanidins quantified individually in this material.

### Putative candidate genes identification

Identification of candidate genes underlying QTLs remains often complicated and time-consuming. Using data from the apple genome sequence, it was possible to screen a first set of candidate genes including enzymes involved in the biosynthesis of phenolic compounds and few transcription factors. This approach, very selective, allowed us to identify several interesting candidates that remain to be validated. Among them, we identified and mapped four genes homologous to *shikimate/quinate O-hydroxycinnamoyl transferase* (*HCT/HQT*) under the QTL confidence interval for the 5-caffeoylquinic acid on the LG17. Two of them were assigned with a very high significance by BLAST (min e-value of 0). The presence of a major gene like *HCT/HQT* controlling the synthesis of 5-caffeoylquinic acid seemed particularly relevant since this region was detected both in female and male maps, with very high proportion of explained phenotypic variation. *HCT/HQT* genes were described to catalyze the formation of *p*-coumaroylquinic acid from the 4-coumaroyl-CoA (Figure 1) [15]. This compound is the precursor of caffeoylquinic acid *via* the *p*-coumarate 3′-hydroxylase (C3'H). Then, caffeoylquinic acid may be used as a substrate by HCT to form caffeoyl-CoA. Conversely, caffeoyl-CoA can be used by HQT to form caffeoylquinic acid (Figure 1) [20]. Because they are directly related to this compound, and considering the high proportion of variability explained by these QTLs, *HCT/HQT* genes are very good candidates.

Similarly, a gene homologous to *flavonoid 3′-hydroxylase* (*F3'H*), responsible for the hydroxylation on the third position of the B ring of flavonols, dihydroflavonols or flavanones, was identified and mapped under the quercetin glycosides cluster on LG1 (Figures 1 and 2). If this enzyme is up-regulated, the formation of quercetin could be favored compared to kaempferol (compound for which QTLs were detected on LG1 in the Khan *et al*. study [35]). Inversely, if it is down-regulated, the degradation of kaempferol is lower and this class of compounds could be favored compared to quercetin. However, *F3'H* genes belong to a multigenic family and validation tests are needed to confirm the role of the *F3'H* gene identified in the confidence interval of this QTL in the biosynthesis of flavonols.

A homologue of *UDP-glucose 3-glucosyltransferase* (*UFGT*) gene was identified under QTLs for flavanols on LG1. This gene is described to catalyze the formation of anthocyanidins-3-*O*-β-*D*-glucoside from anthocyanidins and UDP-*D*-glucose (Figures 1 and 2). This colocation with flavanol QTLs may be explained by the competition of this enzyme with anthocyanidin reductase (ANR) for anthocyanidins as a substrate to form either cyanidin glycosides (with UFGT) or flavanol monomers (catechin and epicatechin). Indeed, ectopic expression of apple *MdANR* genes in tobacco increases the procyanidins content and decreases the anthocyanin content in flowers [59]. In the same way, the silencing of *anthocyanidin synthase* (*ANS*) in apple has shown a drastic reduction in the anthocyanin content [60]. However, in this last study, the epicatechin content was increased whereas this compound is also dependent of the ANS activity. Authors supposed a residual ANS activity associated with kinetic competition between ANR and UFGT. An alternative biosynthetic pathway to epicatechin from catechin or procyanidins was also suggested [60]. The studies of Han *et al*. [59] and Szankowski *et al*. [60] have also shown a modification in the transcriptional level and/or in the enzymatic activities of almost all structural enzymes of the polyphenolic pathway, as well as the MYB transcription factor in tobacco. These results suggest a complex feedback of biosynthetic enzymes that remains to be clarified to evaluate clearly the impact of each enzyme on the polyphenol pathway.

Transcription factors involved in the regulation of phenolic compounds in apple have been much less studied. A MYB gene located on LG9 (with three alleles *MYB1* [28]/*MYBA* [29]/*MYB10* [30]), two other MYB genes called *MYB110a* and *MYB110b* located on LG17 [31], two bHLH (*MdbHLH3* on LG11 [32] and *MdbHLH33* on LG15 [30], [33]) and a WD40 (*MdTTG1* on LG1 [33]) transcription factors involved in the anthocyanin pathway have been previously identified. In the present study, the clustering of four of these transcription factors (*MdTTG1*, *bHLH33*, *MYB110a* and *MYB110b*) with regions grouping many QTLs detected for several phenolic groups (hydroxycinnamic acids and flavanols on LG1, flavanols and flavonols on LG15 and hydroxycinnamic acids and flavonols on LG17) suggests a more extended involvement of these transcription factors in gene regulation, not only restricted to anthocyanins pathway (Figure 2).

This candidate gene mapping approach was based on an *in silico* identification and a genetic mapping of genes potentially involved in the polyphenol pathway and its regulation. Focusing on a limited number of genes, some other possible functions remain to be investigated, like additional transcription factors, small regulatory RNA (miRNA) and other genes which could affect the catalytic activity of enzymes or phenolic compounds transport or stability. However, this study contributed to highlight a large number of candidate genes for most of the major QTLs. It also highlights the complexity of the biosynthesis of these compounds by showing the absence of major expected gene of the biosynthesis under major QTLs like the *ANR* on LG16 and the *HCT/HQT* on the LG17. Following a fine mapping approach of the QTL of interest, a functional validation can now be undertaken, using sequencing methods, QRT-PCR, and transgenesis. QTL detection based on the level of expression of these genes (eQTL) would also permit further in depth understanding of the phenolic compounds biosynthesis.

### Potential use in marker assisted selection

This study has opened new ways for breeders to select new varieties with specific phenolic compounds affecting the taste of cider. QTLs detected on LG1 for flavanols, LG5 for dihydrochalcones, LG15 for flavonols and LG16 for DPn show high stability between years and materials (fruit and juice), with high proportion of explained phenotypic variation. QTLs detected in this study on LG14 and LG17 for hydroxycinnamic acids were also identified in previous studies on dessert apple. Candidate genes identified under these QTLs reinforce their interest for breeding programs.

## Conclusion

This study is the first performed on a cider apple progeny, highlighting QTLs responsible for the variability of major phenolic compounds involved in cider organoleptic characteristics as well as the main polymerization degree of procyanidins. These QTLs are the first detected in apple and represent a new step to understand the mechanism of procyanidin biosynthesis, which appears to be independent from the synthesis of flavanols. This study has also confirmed the importance of two regions involved in the biosynthesis of hydroxycinnamic acids on LG14 and LG17. Other important regions were newly detected in this study on LG1, LG5 and LG15 for flavanols, dihydrochalcones and flavonols, respectively. Moreover, the identification of candidate genes performed *in silico* has shown interesting targets for future studies aiming to better understand the biosynthesis of phenolic compounds.

## Supporting Information

Figure S1 Ancestors of the progeny studied.
(PDF)

Figure S2 Parental genetic maps for X5210 (A) and X8402 (B) built using JoinMap 4.0. software with SSR and SNP markers.
(PDF)

Table S1 Summary of phenolic compounds quantified in fruit harvested in 2008 (F08) and 2009 (F09) and in juice prepared in 2009 (J09) and 2010 (J10).
(XLSX)

Table S2 SNP markers identified in the ‘Golden Delicious’ genome sequence.
(XLS)
