# Supplementary figures and images for: QTL Analysis and Candidate Gene Mapping for the Polyphenol Content in Cider Apple
Source: PLoS One. 2014 Oct 1;9(10):e107103. doi: 10.1371/journal.pone.0107103 (PMC4182701; doi:10.1371/journal.pone.0107103)

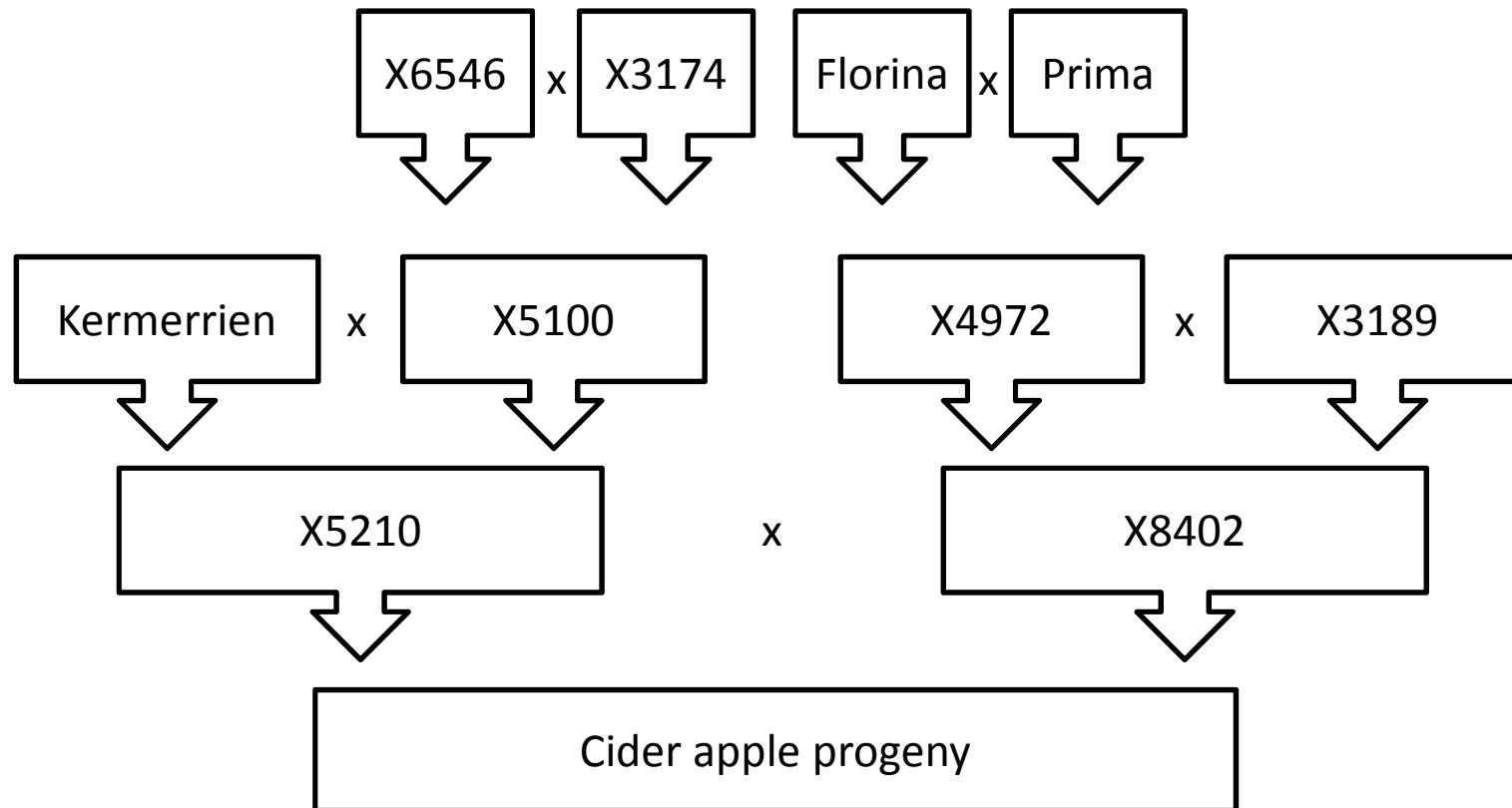

Supplement: Figure S1 — Ancestors of the progeny studied. (PDF) [file pone.0107103.s001.pdf]

A)

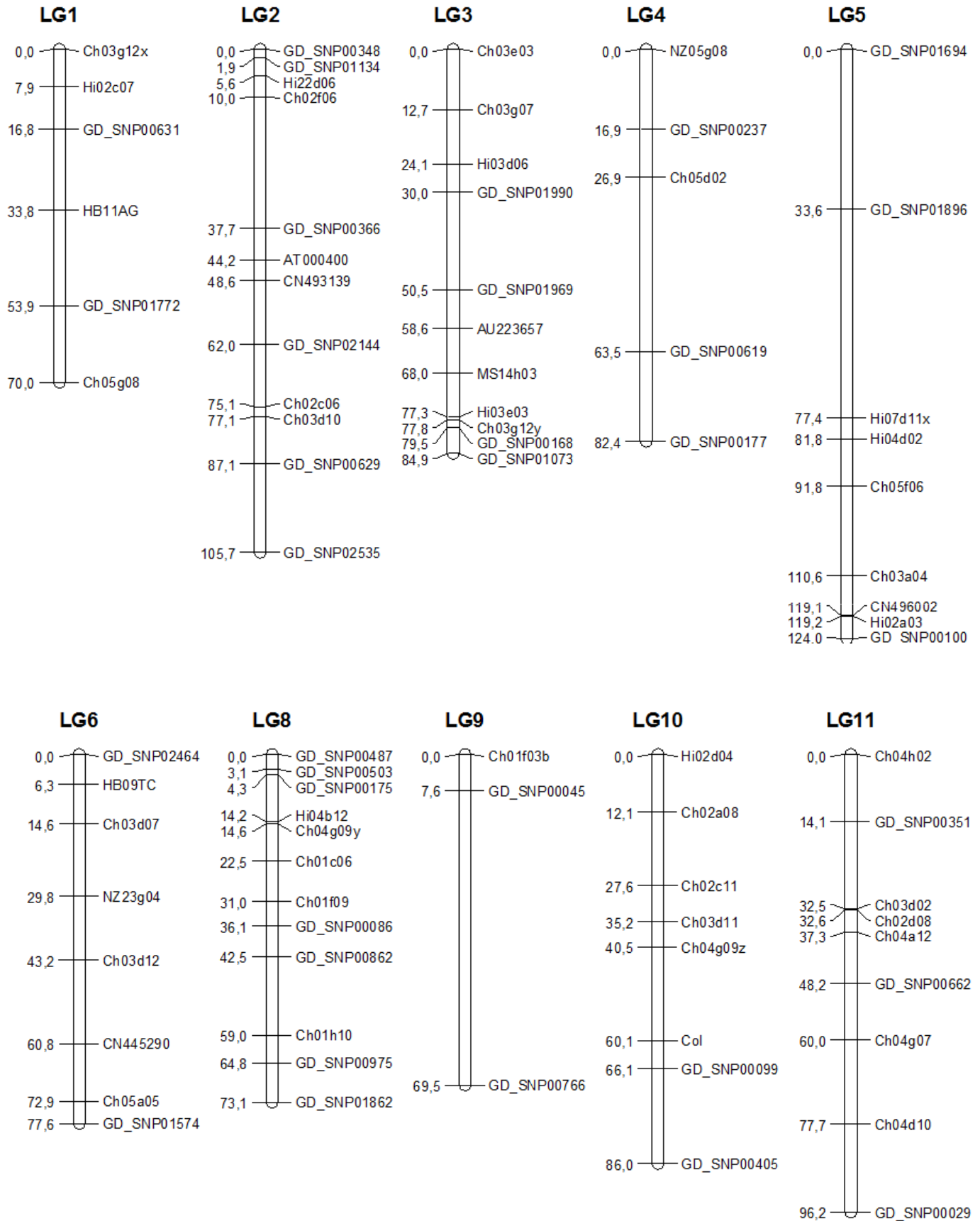

**LG12**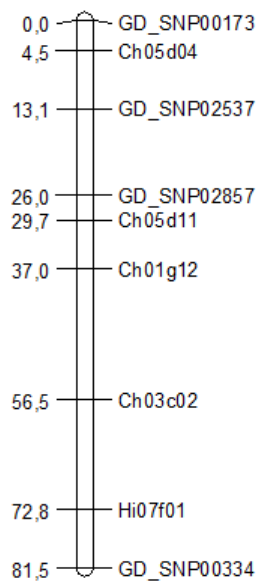**LG13**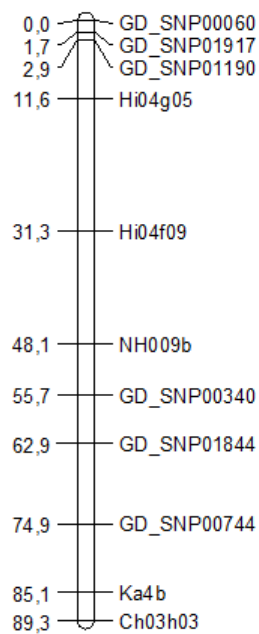**LG14**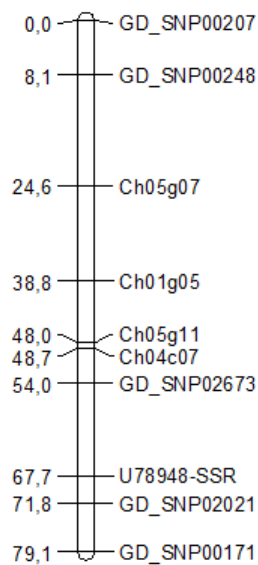**LG15**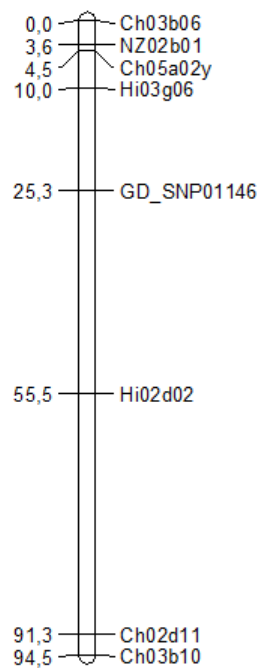**LG16**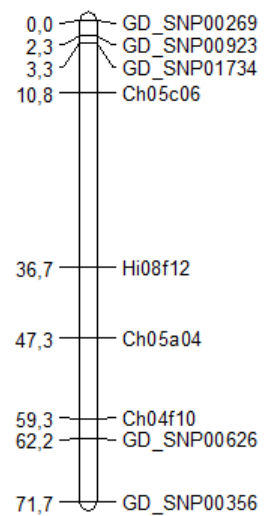**LG17**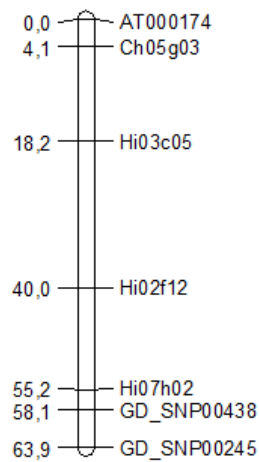

B)

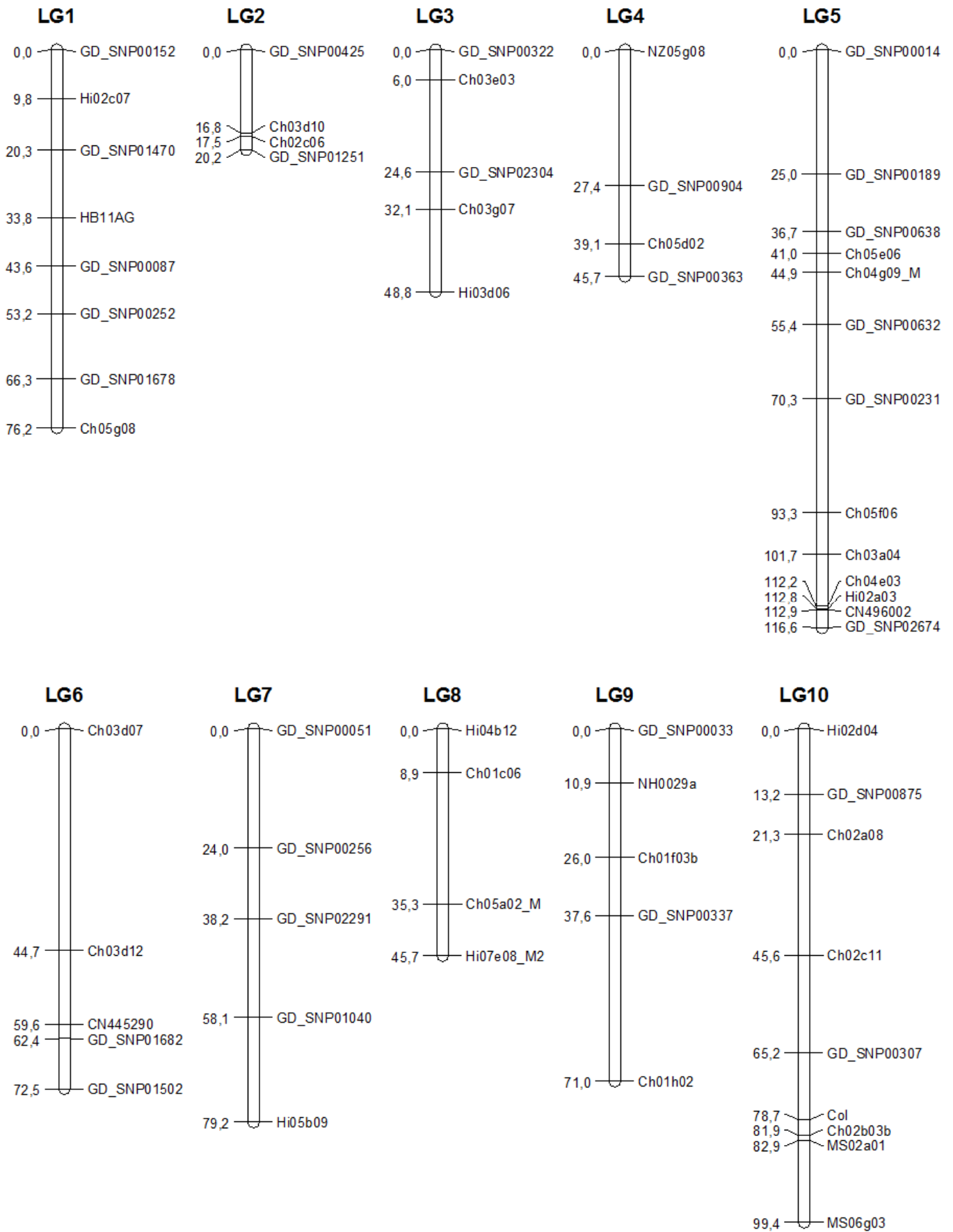

**LG11**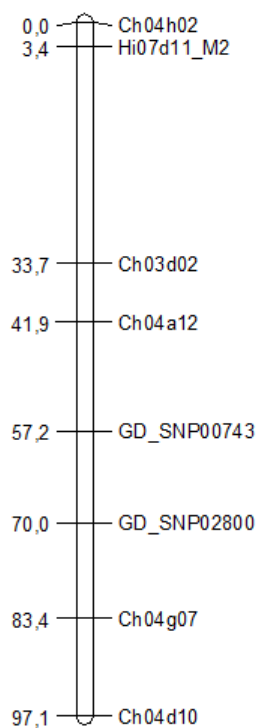**LG12**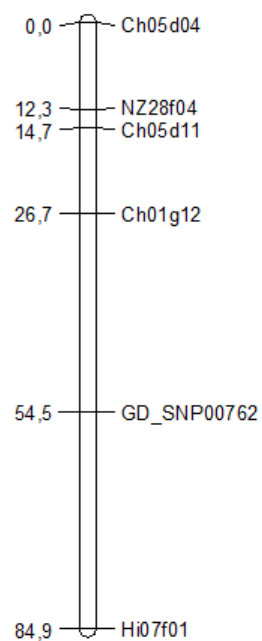**LG13**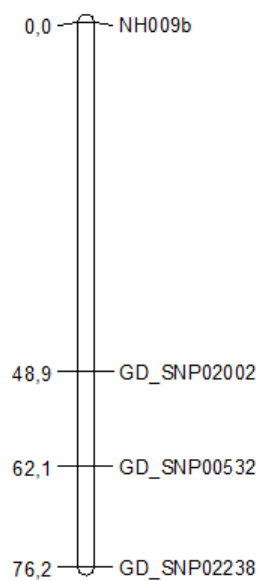**LG14**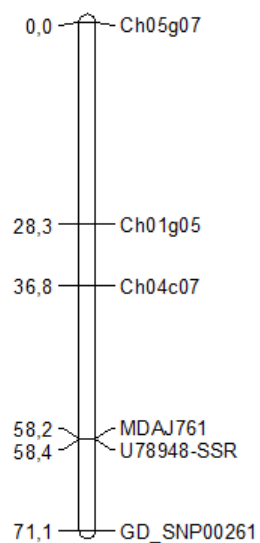**LG15**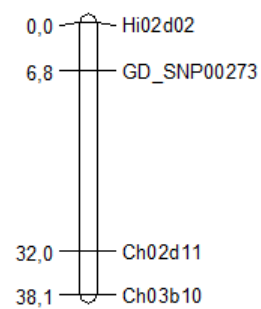**LG16**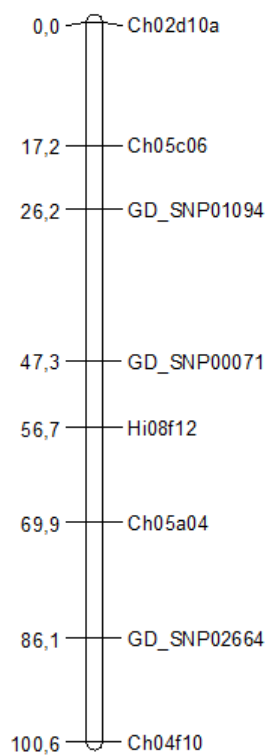**LG17**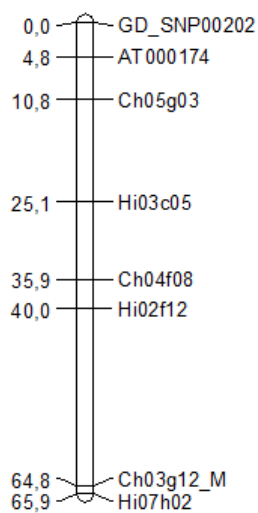

Supplement: Figure S2 — Parental genetic maps for X5210 (A) and X8402 (B) built using JoinMap 4.0. software with SSR and SNP markers. (PDF) [file pone.0107103.s002.pdf]
